# Neutrophil gelatinase-associated lipocalin predicts postoperative fluid overload after cardiac surgery

**DOI:** 10.1186/cc12364

**Published:** 2013-03-19

**Authors:** M Haase, P Devarajan, P Michael, R Bellomo, A Haase-Fielitz

**Affiliations:** 1 Otto von-Guericke University, Magdeburg, Germany; 2 Cincinnati Children's Hospital Medical Center, Cincinnati, OH, USA; 3 German Heart Center, Berlin, Germany; 4 Austin Hospital, Melbourne, Australia

## Introduction

Neutrophil gelatinase-associated lipocalin (NGAL), measured early after cardiac surgery, has been demonstrated to predict postoperative acute kidney injury (AKI). Fluid overload potentially masks a subsequent acute renal function loss through dilution of serum creatinine and maintenance of urine output just above AKI-defining criteria.

## Methods

We investigated the early postoperative value of NGAL versus that of simultaneously measured serum creatinine to predict subsequent fluid overload. We studied 100 adult cardiac surgery patients in the control arm of a RCT (NCT00672334). Severe postoperative fluid overload was defined as positive fluid balance >10% of preoperative body weight within 48 hours after surgery.

## Results

**Figure 1:**
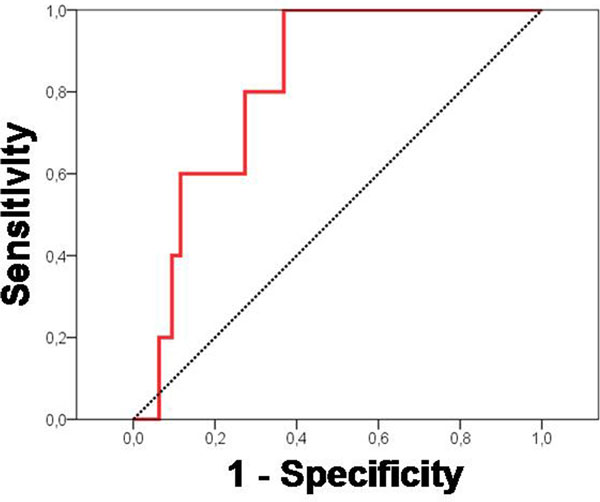
**NGAL at ICU admission predicts fluid overload of >10% of body weight**.

Severe postoperative fluid overload was present in 5% of patients with a mean positive fluid balance of 15.8 ± 9.5 l. At ICU admission, urine NGAL predicted severe fluid overload (AUC-ROC 0.82 (95% CI = 0.70 to 0.94)) (Figure 1) and mortality (AUC 0.88 (0.78 to 0.97)). Serum creatinine measured at the same time did not predict severe fluid overload (AUC 0.52 (0.26 to 0.79)) or mortality (AUC 0.61 (0.16 to 0.99)).

## Conclusion

Early NGAL-guided adjustments to fluid management may reduce organ edema after cardiac surgery. Findings should be validated in larger cohorts.

